# Supplementary material for: Modeling glioblastoma heterogeneity as a dynamic network of cell states
Source: Mol Syst Biol. 2021 Sep 16;17(9):e10105. doi: 10.15252/msb.202010105 (PMC8444284; doi:10.15252/msb.202010105)
Supplement: Supplementary file 6 — Source Data for Figure 5 [file MSB-17-e10105-s004.zip › Figure5A_sourcedata/GSEA_3071/nosigdb_stateIV.GseaPreranked.1622795164626/FELDMAN_2020_S-G2.html]

Details for gene set FELDMAN\_2020\_S-G2[GSEA]

|  || Dataset | state53071 |
| Phenotype | NoPhenotypeAvailable |
| Upregulated in class | na\_neg |
| GeneSet | FELDMAN\_2020\_S-G2 |
| Enrichment Score (ES) | -0.4877234 |
| Normalized Enrichment Score (NES) | -3.0014637 |
| Nominal p-value | 0.0 |
| FDR q-value | 0.0 |
| FWER p-Value | 0.0 |
Table: GSEA Results Summary

  

Fig 1: Enrichment plot: FELDMAN\_2020\_S-G2      
 Profile of the Running ES Score & Positions of GeneSet Members on the Rank Ordered List

  

| PROBE | GENE SYMBOL | GENE\_TITLE | RANK IN GENE LIST | RANK METRIC SCORE | RUNNING ES | CORE ENRICHMENT || 1 | PTPRZ1 |  |  | 10 | 2.539 | 0.0092 | No |
| 2 | S100B |  |  | 12 | 2.461 | 0.0198 | No |
| 3 | FABP7 |  |  | 14 | 2.398 | 0.0302 | No |
| 4 | BEX1 |  |  | 18 | 2.252 | 0.0395 | No |
| 5 | GPM6B |  |  | 21 | 2.210 | 0.0489 | No |
| 6 | CLU |  |  | 27 | 2.064 | 0.0570 | No |
| 7 | SCRG1 |  |  | 28 | 2.056 | 0.0660 | No |
| 8 | IGFBP3 |  |  | 59 | 1.794 | 0.0680 | No |
| 9 | CDH2 |  |  | 68 | 1.735 | 0.0740 | No |
| 10 | GPM6A |  |  | 98 | 1.538 | 0.0750 | No |
| 11 | PCSK1N |  |  | 110 | 1.507 | 0.0795 | No |
| 12 | TM7SF2 |  |  | 113 | 1.500 | 0.0857 | No |
| 13 | TUBA1A |  |  | 165 | 1.337 | 0.0814 | No |
| 14 | SOX9 |  |  | 214 | 1.223 | 0.0772 | No |
| 15 | ITM2B |  |  | 233 | 1.181 | 0.0789 | No |
| 16 | PRCP |  |  | 242 | 1.169 | 0.0824 | No |
| 17 | PLAT |  |  | 281 | 1.070 | 0.0796 | No |
| 18 | LYPD1 |  |  | 338 | 0.980 | 0.0727 | No |
| 19 | BEX4 |  |  | 341 | 0.980 | 0.0766 | No |
| 20 | ZFAND6 |  |  | 344 | 0.978 | 0.0805 | No |
| 21 | SOX4 |  |  | 377 | 0.928 | 0.0783 | No |
| 22 | SESN3 |  |  | 380 | 0.925 | 0.0819 | No |
| 23 | TTYH1 |  |  | 404 | 0.906 | 0.0813 | No |
| 24 | PSAP |  |  | 501 | 0.816 | 0.0658 | No |
| 25 | SCD5 |  |  | 555 | 0.759 | 0.0586 | No |
| 26 | ENC1 |  |  | 590 | 0.734 | 0.0551 | No |
| 27 | NDFIP1 |  |  | 618 | 0.714 | 0.0528 | No |
| 28 | RNF19A |  |  | 649 | 0.692 | 0.0499 | No |
| 29 | MALAT1 |  |  | 739 | 0.638 | 0.0350 | No |
| 30 | SAT1 |  |  | 859 | 0.575 | 0.0138 | No |
| 31 | EIF4A2 |  |  | 860 | 0.575 | 0.0163 | No |
| 32 | SYNE2 |  |  | 1094 | 0.493 | -0.0279 | No |
| 33 | BTG1 |  |  | 1123 | 0.486 | -0.0314 | No |
| 34 | TMEM158 |  |  | 1588 | 0.369 | -0.1222 | No |
| 35 | WDR83OS |  |  | 1761 | 0.337 | -0.1550 | No |
| 36 | TMEM178A |  |  | 1823 | 0.327 | -0.1657 | No |
| 37 | PLD3 |  |  | 1913 | 0.312 | -0.1821 | No |
| 38 | MLLT11 |  |  | 1932 | 0.308 | -0.1843 | No |
| 39 | ATP6V1G1 |  |  | 2148 | 0.277 | -0.2259 | No |
| 40 | SNRNP25 |  |  | 2149 | 0.277 | -0.2247 | No |
| 41 | RABAC1 |  |  | 2169 | 0.272 | -0.2273 | No |
| 42 | CARHSP1 |  |  | 2185 | 0.270 | -0.2291 | No |
| 43 | PIN1 |  |  | 2204 | 0.267 | -0.2315 | No |
| 44 | PTTG1 |  |  | 2470 | -0.266 | -0.2832 | No |
| 45 | LMNB1 |  |  | 2487 | -0.268 | -0.2852 | No |
| 46 | EIF1AX |  |  | 2584 | -0.280 | -0.3031 | No |
| 47 | RNASEH2A |  |  | 2595 | -0.282 | -0.3038 | No |
| 48 | CDKN2D |  |  | 2615 | -0.284 | -0.3063 | No |
| 49 | CMC2 |  |  | 2632 | -0.287 | -0.3083 | No |
| 50 | POP7 |  |  | 2651 | -0.290 | -0.3106 | No |
| 51 | POLD4 |  |  | 2676 | -0.292 | -0.3141 | No |
| 52 | TMEM237 |  |  | 2811 | -0.307 | -0.3394 | No |
| 53 | PRKDC |  |  | 2815 | -0.307 | -0.3387 | No |
| 54 | HMGB1 |  |  | 2819 | -0.308 | -0.3379 | No |
| 55 | ARL6IP6 |  |  | 2877 | -0.316 | -0.3479 | No |
| 56 | TNFRSF12A |  |  | 2890 | -0.318 | -0.3489 | No |
| 57 | RMI2 |  |  | 2893 | -0.318 | -0.3479 | No |
| 58 | RRS1 |  |  | 2898 | -0.319 | -0.3473 | No |
| 59 | DCK |  |  | 2907 | -0.319 | -0.3474 | No |
| 60 | HNRNPAB |  |  | 2944 | -0.324 | -0.3532 | No |
| 61 | SMC1A |  |  | 2968 | -0.328 | -0.3563 | No |
| 62 | SMC4 |  |  | 3035 | -0.337 | -0.3680 | No |
| 63 | SNRPG |  |  | 3085 | -0.343 | -0.3762 | No |
| 64 | HAUS1 |  |  | 3106 | -0.347 | -0.3787 | No |
| 65 | ACYP1 |  |  | 3220 | -0.363 | -0.3996 | No |
| 66 | MXD3 |  |  | 3316 | -0.377 | -0.4169 | No |
| 67 | CISD2 |  |  | 3319 | -0.378 | -0.4156 | No |
| 68 | HIRIP3 |  |  | 3328 | -0.379 | -0.4155 | No |
| 69 | TUBA1B |  |  | 3357 | -0.383 | -0.4194 | No |
| 70 | DCTPP1 |  |  | 3414 | -0.393 | -0.4289 | No |
| 71 | TFDP1 |  |  | 3526 | -0.415 | -0.4492 | No |
| 72 | MRPL18 |  |  | 3568 | -0.422 | -0.4555 | No |
| 73 | PAICS |  |  | 3594 | -0.427 | -0.4586 | No |
| 74 | CKAP2 |  |  | 3633 | -0.433 | -0.4642 | No |
| 75 | SNRPA1 |  |  | 3638 | -0.434 | -0.4631 | No |
| 76 | TMEM106C |  |  | 3669 | -0.438 | -0.4672 | No |
| 77 | HSPA8 |  |  | 3720 | -0.449 | -0.4752 | No |
| 78 | PGP |  |  | 3725 | -0.450 | -0.4740 | No |
| 79 | BAMBI |  |  | 3767 | -0.458 | -0.4801 | No |
| 80 | TUBG1 |  |  | 3775 | -0.460 | -0.4795 | No |
| 81 | HES4 |  |  | 3779 | -0.461 | -0.4781 | No |
| 82 | DERA |  |  | 3799 | -0.466 | -0.4798 | No |
| 83 | FBL |  |  | 3819 | -0.469 | -0.4815 | No |
| 84 | KIF22 |  |  | 3832 | -0.472 | -0.4818 | No |
| 85 | HSPD1 |  |  | 3842 | -0.474 | -0.4815 | No |
| 86 | CMTM7 |  |  | 3869 | -0.480 | -0.4846 | No |
| 87 | SUPT16H |  |  | 3872 | -0.481 | -0.4829 | No |
| 88 | SNRPE |  |  | 3878 | -0.482 | -0.4817 | No |
| 89 | CALM2 |  |  | 3909 | -0.489 | -0.4856 | Yes |
| 90 | PARP2 |  |  | 3910 | -0.490 | -0.4834 | Yes |
| 91 | MIS18BP1 |  |  | 3913 | -0.490 | -0.4816 | Yes |
| 92 | RAB13 |  |  | 3933 | -0.494 | -0.4833 | Yes |
| 93 | SMC3 |  |  | 3952 | -0.498 | -0.4847 | Yes |
| 94 | PTGES3 |  |  | 3953 | -0.498 | -0.4825 | Yes |
| 95 | PRELID1 |  |  | 3971 | -0.504 | -0.4836 | Yes |
| 96 | MAGOHB |  |  | 3992 | -0.509 | -0.4854 | Yes |
| 97 | CENPF |  |  | 4003 | -0.511 | -0.4851 | Yes |
| 98 | ARHGAP11A |  |  | 4007 | -0.513 | -0.4834 | Yes |
| 99 | HIST1H1E |  |  | 4011 | -0.515 | -0.4818 | Yes |
| 100 | WDR34 |  |  | 4022 | -0.518 | -0.4815 | Yes |
| 101 | HSP90AA1 |  |  | 4024 | -0.519 | -0.4794 | Yes |
| 102 | TOMM40 |  |  | 4030 | -0.520 | -0.4781 | Yes |
| 103 | YWHAH |  |  | 4032 | -0.521 | -0.4760 | Yes |
| 104 | DCBLD2 |  |  | 4038 | -0.521 | -0.4747 | Yes |
| 105 | CYCS |  |  | 4074 | -0.532 | -0.4793 | Yes |
| 106 | TEX30 |  |  | 4076 | -0.532 | -0.4772 | Yes |
| 107 | ZWILCH |  |  | 4082 | -0.534 | -0.4758 | Yes |
| 108 | TUBB |  |  | 4083 | -0.534 | -0.4735 | Yes |
| 109 | BRIX1 |  |  | 4106 | -0.542 | -0.4755 | Yes |
| 110 | CACYBP |  |  | 4109 | -0.542 | -0.4735 | Yes |
| 111 | SSRP1 |  |  | 4112 | -0.544 | -0.4715 | Yes |
| 112 | NXT1 |  |  | 4170 | -0.560 | -0.4804 | Yes |
| 113 | NCL |  |  | 4171 | -0.560 | -0.4779 | Yes |
| 114 | HPRT1 |  |  | 4179 | -0.562 | -0.4768 | Yes |
| 115 | SNRNP40 |  |  | 4180 | -0.562 | -0.4743 | Yes |
| 116 | MRPL17 |  |  | 4210 | -0.569 | -0.4776 | Yes |
| 117 | PSMC3 |  |  | 4224 | -0.573 | -0.4777 | Yes |
| 118 | MIS18A |  |  | 4230 | -0.575 | -0.4761 | Yes |
| 119 | ANP32B |  |  | 4235 | -0.576 | -0.4744 | Yes |
| 120 | HMGB2 |  |  | 4243 | -0.578 | -0.4733 | Yes |
| 121 | BUB3 |  |  | 4249 | -0.580 | -0.4717 | Yes |
| 122 | SNRPD1 |  |  | 4254 | -0.582 | -0.4699 | Yes |
| 123 | SIVA1 |  |  | 4263 | -0.586 | -0.4689 | Yes |
| 124 | IGFBP5 |  |  | 4280 | -0.592 | -0.4695 | Yes |
| 125 | EXOSC8 |  |  | 4293 | -0.596 | -0.4693 | Yes |
| 126 | SLC25A5 |  |  | 4296 | -0.597 | -0.4671 | Yes |
| 127 | ASF1B |  |  | 4328 | -0.606 | -0.4706 | Yes |
| 128 | RFC4 |  |  | 4340 | -0.611 | -0.4701 | Yes |
| 129 | FTL |  |  | 4350 | -0.615 | -0.4691 | Yes |
| 130 | KPNA2 |  |  | 4361 | -0.621 | -0.4684 | Yes |
| 131 | RBM17 |  |  | 4362 | -0.622 | -0.4657 | Yes |
| 132 | RBBP8 |  |  | 4375 | -0.627 | -0.4653 | Yes |
| 133 | DNMT1 |  |  | 4388 | -0.632 | -0.4649 | Yes |
| 134 | RHEB |  |  | 4416 | -0.641 | -0.4674 | Yes |
| 135 | CEP78 |  |  | 4420 | -0.642 | -0.4652 | Yes |
| 136 | C19orf48 |  |  | 4427 | -0.644 | -0.4636 | Yes |
| 137 | DONSON |  |  | 4444 | -0.652 | -0.4639 | Yes |
| 138 | SAC3D1 |  |  | 4462 | -0.660 | -0.4644 | Yes |
| 139 | SHCBP1 |  |  | 4473 | -0.667 | -0.4634 | Yes |
| 140 | EIF4EBP1 |  |  | 4474 | -0.667 | -0.4605 | Yes |
| 141 | RPA3 |  |  | 4482 | -0.670 | -0.4589 | Yes |
| 142 | PDCD5 |  |  | 4485 | -0.672 | -0.4564 | Yes |
| 143 | H2AFX |  |  | 4495 | -0.677 | -0.4552 | Yes |
| 144 | PA2G4 |  |  | 4497 | -0.678 | -0.4524 | Yes |
| 145 | NUDT15 |  |  | 4506 | -0.684 | -0.4510 | Yes |
| 146 | DHFR |  |  | 4517 | -0.688 | -0.4499 | Yes |
| 147 | MND1 |  |  | 4538 | -0.699 | -0.4508 | Yes |
| 148 | RFC5 |  |  | 4543 | -0.701 | -0.4485 | Yes |
| 149 | EIF2S1 |  |  | 4553 | -0.707 | -0.4472 | Yes |
| 150 | DTYMK |  |  | 4566 | -0.715 | -0.4465 | Yes |
| 151 | NCAPG |  |  | 4568 | -0.716 | -0.4435 | Yes |
| 152 | DBF4 |  |  | 4570 | -0.717 | -0.4405 | Yes |
| 153 | TUBB4B |  |  | 4575 | -0.722 | -0.4382 | Yes |
| 154 | FHL2 |  |  | 4576 | -0.723 | -0.4350 | Yes |
| 155 | PFDN2 |  |  | 4580 | -0.726 | -0.4324 | Yes |
| 156 | RFC2 |  |  | 4588 | -0.729 | -0.4306 | Yes |
| 157 | UPP1 |  |  | 4591 | -0.729 | -0.4277 | Yes |
| 158 | PBK |  |  | 4593 | -0.730 | -0.4247 | Yes |
| 159 | CKS2 |  |  | 4600 | -0.733 | -0.4227 | Yes |
| 160 | SPDL1 |  |  | 4617 | -0.741 | -0.4226 | Yes |
| 161 | BIRC5 |  |  | 4618 | -0.745 | -0.4193 | Yes |
| 162 | CKLF |  |  | 4619 | -0.746 | -0.4160 | Yes |
| 163 | PTMA |  |  | 4620 | -0.746 | -0.4128 | Yes |
| 164 | MIAT |  |  | 4622 | -0.748 | -0.4097 | Yes |
| 165 | DUT |  |  | 4642 | -0.762 | -0.4101 | Yes |
| 166 | HIST1H2AH |  |  | 4645 | -0.762 | -0.4071 | Yes |
| 167 | USP1 |  |  | 4649 | -0.763 | -0.4044 | Yes |
| 168 | NUSAP1 |  |  | 4654 | -0.765 | -0.4018 | Yes |
| 169 | GGCT |  |  | 4666 | -0.772 | -0.4006 | Yes |
| 170 | CENPH |  |  | 4679 | -0.786 | -0.3995 | Yes |
| 171 | KPNB1 |  |  | 4681 | -0.786 | -0.3962 | Yes |
| 172 | CENPA |  |  | 4683 | -0.788 | -0.3930 | Yes |
| 173 | ID3 |  |  | 4686 | -0.788 | -0.3899 | Yes |
| 174 | COPS3 |  |  | 4694 | -0.791 | -0.3878 | Yes |
| 175 | CDC45 |  |  | 4702 | -0.798 | -0.3857 | Yes |
| 176 | MCM7 |  |  | 4704 | -0.800 | -0.3824 | Yes |
| 177 | SAE1 |  |  | 4711 | -0.801 | -0.3800 | Yes |
| 178 | TCF19 |  |  | 4719 | -0.807 | -0.3779 | Yes |
| 179 | TRIP13 |  |  | 4721 | -0.808 | -0.3745 | Yes |
| 180 | GTSE1 |  |  | 4722 | -0.808 | -0.3709 | Yes |
| 181 | KIF4A |  |  | 4727 | -0.812 | -0.3682 | Yes |
| 182 | CDCA2 |  |  | 4732 | -0.816 | -0.3654 | Yes |
| 183 | RANBP1 |  |  | 4741 | -0.823 | -0.3633 | Yes |
| 184 | RAN |  |  | 4743 | -0.824 | -0.3599 | Yes |
| 185 | KIF20B |  |  | 4745 | -0.825 | -0.3565 | Yes |
| 186 | CENPE |  |  | 4757 | -0.832 | -0.3550 | Yes |
| 187 | MELK |  |  | 4758 | -0.835 | -0.3513 | Yes |
| 188 | FOSL1 |  |  | 4761 | -0.836 | -0.3480 | Yes |
| 189 | CKAP2L |  |  | 4769 | -0.839 | -0.3457 | Yes |
| 190 | VRK1 |  |  | 4773 | -0.842 | -0.3426 | Yes |
| 191 | NUF2 |  |  | 4782 | -0.847 | -0.3405 | Yes |
| 192 | PRDX1 |  |  | 4783 | -0.848 | -0.3367 | Yes |
| 193 | ASPM |  |  | 4789 | -0.851 | -0.3340 | Yes |
| 194 | TMPO |  |  | 4790 | -0.851 | -0.3302 | Yes |
| 195 | CENPN |  |  | 4796 | -0.858 | -0.3274 | Yes |
| 196 | HIST2H2AC |  |  | 4801 | -0.866 | -0.3244 | Yes |
| 197 | LRR1 |  |  | 4805 | -0.873 | -0.3212 | Yes |
| 198 | HELLS |  |  | 4819 | -0.882 | -0.3199 | Yes |
| 199 | HSPB11 |  |  | 4820 | -0.882 | -0.3160 | Yes |
| 200 | KIF23 |  |  | 4825 | -0.891 | -0.3129 | Yes |
| 201 | MCM3 |  |  | 4834 | -0.901 | -0.3105 | Yes |
| 202 | MKI67 |  |  | 4836 | -0.902 | -0.3067 | Yes |
| 203 | KIFC1 |  |  | 4844 | -0.911 | -0.3041 | Yes |
| 204 | PRC1 |  |  | 4846 | -0.912 | -0.3003 | Yes |
| 205 | SMC2 |  |  | 4847 | -0.912 | -0.2962 | Yes |
| 206 | CTGF |  |  | 4852 | -0.915 | -0.2930 | Yes |
| 207 | DSN1 |  |  | 4864 | -0.929 | -0.2911 | Yes |
| 208 | ORC6 |  |  | 4865 | -0.931 | -0.2870 | Yes |
| 209 | GMNN |  |  | 4873 | -0.939 | -0.2843 | Yes |
| 210 | CDCA3 |  |  | 4874 | -0.940 | -0.2801 | Yes |
| 211 | PMAIP1 |  |  | 4875 | -0.940 | -0.2760 | Yes |
| 212 | HIST1H1C |  |  | 4877 | -0.940 | -0.2720 | Yes |
| 213 | CENPM |  |  | 4881 | -0.947 | -0.2685 | Yes |
| 214 | GINS2 |  |  | 4897 | -0.962 | -0.2672 | Yes |
| 215 | TACC3 |  |  | 4900 | -0.966 | -0.2633 | Yes |
| 216 | CDCA4 |  |  | 4902 | -0.967 | -0.2593 | Yes |
| 217 | RTKN2 |  |  | 4904 | -0.970 | -0.2552 | Yes |
| 218 | FBXO5 |  |  | 4910 | -0.978 | -0.2519 | Yes |
| 219 | SKA3 |  |  | 4914 | -0.981 | -0.2482 | Yes |
| 220 | DNAJC9 |  |  | 4916 | -0.982 | -0.2440 | Yes |
| 221 | H2AFZ |  |  | 4919 | -0.983 | -0.2401 | Yes |
| 222 | SNRPB |  |  | 4931 | -0.996 | -0.2379 | Yes |
| 223 | PSMC3IP |  |  | 4933 | -0.997 | -0.2337 | Yes |
| 224 | ATAD5 |  |  | 4934 | -0.998 | -0.2293 | Yes |
| 225 | TUBB6 |  |  | 4939 | -1.003 | -0.2257 | Yes |
| 226 | CTNNAL1 |  |  | 4945 | -1.007 | -0.2223 | Yes |
| 227 | KLF6 |  |  | 4953 | -1.024 | -0.2191 | Yes |
| 228 | PSMG1 |  |  | 4955 | -1.025 | -0.2148 | Yes |
| 229 | CEP55 |  |  | 4972 | -1.047 | -0.2134 | Yes |
| 230 | CENPW |  |  | 4978 | -1.056 | -0.2097 | Yes |
| 231 | ATAD2 |  |  | 4980 | -1.058 | -0.2053 | Yes |
| 232 | RRM1 |  |  | 4990 | -1.074 | -0.2023 | Yes |
| 233 | UBE2T |  |  | 5000 | -1.091 | -0.1993 | Yes |
| 234 | EIF5 |  |  | 5006 | -1.105 | -0.1955 | Yes |
| 235 | MAD2L1 |  |  | 5007 | -1.105 | -0.1906 | Yes |
| 236 | SPC25 |  |  | 5012 | -1.112 | -0.1865 | Yes |
| 237 | FEN1 |  |  | 5014 | -1.113 | -0.1818 | Yes |
| 238 | AURKB |  |  | 5016 | -1.118 | -0.1771 | Yes |
| 239 | TK1 |  |  | 5020 | -1.126 | -0.1727 | Yes |
| 240 | TOP2A |  |  | 5029 | -1.152 | -0.1692 | Yes |
| 241 | MYBL2 |  |  | 5041 | -1.172 | -0.1662 | Yes |
| 242 | TPX2 |  |  | 5049 | -1.184 | -0.1624 | Yes |
| 243 | CCNA2 |  |  | 5051 | -1.186 | -0.1574 | Yes |
| 244 | DEPDC1 |  |  | 5053 | -1.193 | -0.1523 | Yes |
| 245 | NDC80 |  |  | 5055 | -1.200 | -0.1472 | Yes |
| 246 | PCNA |  |  | 5060 | -1.214 | -0.1427 | Yes |
| 247 | ESCO2 |  |  | 5067 | -1.222 | -0.1385 | Yes |
| 248 | BRCA2 |  |  | 5069 | -1.222 | -0.1333 | Yes |
| 249 | CENPK |  |  | 5091 | -1.267 | -0.1319 | Yes |
| 250 | UBE2C |  |  | 5106 | -1.314 | -0.1289 | Yes |
| 251 | SMS |  |  | 5109 | -1.315 | -0.1235 | Yes |
| 252 | CCDC34 |  |  | 5111 | -1.316 | -0.1179 | Yes |
| 253 | ZWINT |  |  | 5115 | -1.321 | -0.1127 | Yes |
| 254 | CKS1B |  |  | 5121 | -1.338 | -0.1078 | Yes |
| 255 | HJURP |  |  | 5125 | -1.344 | -0.1025 | Yes |
| 256 | C12orf75 |  |  | 5128 | -1.356 | -0.0969 | Yes |
| 257 | RAD51AP1 |  |  | 5133 | -1.375 | -0.0916 | Yes |
| 258 | SERPINF1 |  |  | 5148 | -1.414 | -0.0882 | Yes |
| 259 | PHLDA2 |  |  | 5155 | -1.458 | -0.0830 | Yes |
| 260 | TYMS |  |  | 5156 | -1.477 | -0.0764 | Yes |
| 261 | CENPU |  |  | 5159 | -1.486 | -0.0703 | Yes |
| 262 | DIAPH3 |  |  | 5161 | -1.488 | -0.0639 | Yes |
| 263 | CDK1 |  |  | 5164 | -1.512 | -0.0577 | Yes |
| 264 | PLP2 |  |  | 5176 | -1.552 | -0.0530 | Yes |
| 265 | ANLN |  |  | 5183 | -1.577 | -0.0473 | Yes |
| 266 | CLSPN |  |  | 5202 | -1.663 | -0.0435 | Yes |
| 267 | IL11 |  |  | 5219 | -1.763 | -0.0390 | Yes |
| 268 | HMGA1 |  |  | 5220 | -1.792 | -0.0311 | Yes |
| 269 | HIST1H1B |  |  | 5223 | -1.811 | -0.0235 | Yes |
| 270 | HIST1H1D |  |  | 5231 | -1.871 | -0.0166 | Yes |
| 271 | RRM2 |  |  | 5232 | -1.875 | -0.0084 | Yes |
| 272 | HIST1H4C |  |  | 5249 | -2.081 | -0.0024 | Yes |
| 273 | RGCC |  |  | 5264 | -2.349 | 0.0052 | Yes |
Table: GSEA details [plain text format]

  

Fig 2: FELDMAN\_2020\_S-G2: Random ES distribution      
 Gene set null distribution of ES for **FELDMAN\_2020\_S-G2**
